# Ultrasound screening for asymptomatic deep vein thrombosis in critically ill patients: a pilot trial

**DOI:** 10.1007/s11739-022-03085-8

**Published:** 2022-08-31

**Authors:** Giordano Tini, Amanda Moriconi, Stefano Ministrini, Valentina Zullo, Elisa Venanzi, Giulia Mondovecchio, Tommaso Campanella, Ettore Marini, Maura Bianchi, Federico Carbone, Matteo Pirro, Edoardo De Robertis, Leonella Pasqualini

**Affiliations:** 1 Internal Medicine, Angiology and Atherosclerosis-Department of Medicine and Surgery, Università Degli Studi Di Perugia, Piazzale Gambuli 1/8, 06124 Perugia, Italy; 2 Medicine Clinic, “S. Lorenzo” Hospital, Viale Vicenza 9, 38051 Borgo Valsugana, TN Italy; 3 Center for Molecular Cardiology, University of Zurich, Wagistrasse 12, 8952 Schlieren, Switzerland; 4 Anesthesia, Analgesia and Intensive Care-Department of Medicine and Surgery, Università Degli Studi Di Perugia, Piazzale Gambuli 1/8, 06124 Perugia, Italy; 5 First Clinic of Internal Medicine, Department of Internal Medicine and Medical Specialties, University of Genoa, 6 Viale Benedetto XV, 16132 Genoa, Italy; 6 IRCCS Ospedale Policlinico San Martino, 10 Largo Rosanna Benzi, 16132 Genoa, Italy

**Keywords:** Deep vein thrombosis, Venous thromboembolism prophylaxis, Critically ill patients, Intensive care, Color-Doppler ultrasound

## Abstract

Deep vein thrombosis (DVT) in critically ill patients still represents a clinical challenge. The aim of the study was to investigate whether a systematic ultrasound (US) screening might improve the management of the antithrombotic therapy in intensive care unit (ICU). In this non-randomized diagnostic clinical trial, 100 patients consecutively admitted to ICU of the University Hospital of Perugia were allocated either in the screening group or in the control group. Subjects in the screening group underwent US examination of lower limbs 48 h after admission, and again after 5 days. Subjects in the control group underwent US examination according to the standard of care (SOC) of the enrolling institution. Retrospectively registered at ClinicalTrials.gov (NCT05019092) on 24.08.2021. Lower limb DVT was significantly more frequent in the screening group (*p* < 0.001), as well as the subsequent extension of a pre-existing DVT (*p* = 0.027). In the control group, DVT of large veins was more frequent (*p* = 0.038). Major bleedings were reported in 5 patients, 4 in the non-screening group and in 1 in the screening group. Patients in the screening group started the antithrombotic treatment later (*p* = 0.038), although the frequency, dose and duration of the treatment were not different between the two groups. The duration of stay in ICU was longer in the screening group (*p* = 0.007). Active screening for DVT is associated with an increased diagnosis of DVT. The screening could be associated with a reduced incidence of proximal DVT and a reduction in the bleeding risk.

**Supplementary Information:**

The online version contains supplementary material available at 10.1007/s11739-022-03085-8.

## Introduction

Deep vein thrombosis (DVT) in critically ill patients still represents a clinical challenge. Despite the standardized use of thromboprophylaxis, its incidence stands at an average of 12.7% [1, 2]. This is due to the simultaneous presence of general risk factors, including age, heart failure, recent surgery, or direct trauma to the limb, as well as specific ones, such as sedation, prolonged immobilization, use of vasopressors or systematic use of central venous catheters [3–5]. In addition, access to intensive care occurs for an extremely heterogeneous range of diseases, often associated with high bleeding risk (e.g., polytrauma, sepsis, and intracranial bleeding), which constitute a contraindication to an adequate anticoagulant prophylaxis [6]. By adding a further layer of complexity, common signs and symptoms of DVT, such as pain and swelling, are often absent or not reported by patients [5]. Since clinical signs are not reliable in this setting, an imaging support for DVT diagnosis is needed. Compression ultrasound (CUS) can be easily performed in most of cases, but it provides information limited to femoral and popliteal veins. Performing a complete examination, which includes color-Doppler and the distal veins, would then reduce the risk of pulmonary thromboembolism, up to 3 months after ultrasound (US) investigation [7]. In line with that, the Ultrasound Consensus Conference of the Society of Radiologists [8] recommends a complete examination for the diagnosis of DVT. Nevertheless, current guidelines do not recommend ultrasonographic (US) screening for DVT in critically ill patients, due to lack of high-quality evidence for a benefit in reducing the rate of thromboembolic complications [9].

The aim of the study was to investigate whether a systematic US screening might improve the management of the antithrombotic therapy in critically ill patients or it may rather lead to a harmful overdiagnosis.

## Materials and methods

### Enrolled subjects

One-hundred patients consecutively admitted to the ICU of the University Hospital of Perugia between January 1st and June 31st 2021 were included in the present trial. Patients enrolled between January 1st and March 31st (*n* = 50) were allocated in the control group, afterward the subjects were allocated in the screening group (*n* = 50).

All patients older than 18 years were included. Exclusion criteria were: duration of stay in ICU < 5 days, positivity to SARS-CoV-2 infection, pregnancy, active malignancy, established DVT or pulmonary embolism at the admission (both symptomatic or asymptomatic), established coagulation disorders, presence of inferior vena cava filter at the admission, patients admitted from/discharged to the ICU of another hospital (Supplementary Fig. ure1). All procedures were conducted in accordance with the 1964 Helsinki declaration and its later amendments. The study was approved by the Ethics Committee of University of Perugia (Protocol number: 2021/8278). Written informed consent form to participate in the study was collected from all patients or legal representatives. Personal data were stored and analyzed anonymously, according to the EU Regulation 2016/679. The trial is registered at ClinicalTrials.gov (NCT05019092). Registration was completed after the enrolment of patients.

### Study design

Subjects included in the screening group underwent US examination of lower limbs 48 h after admission and again after 5 days (7 days after the admission).

Subjects included in the control group underwent US examination if indicated by clinical evaluation of risk factors for DVT in accordance with the standard of care (SOC) of the enrolling institution. To reduce bias, US examinations were performed by two separate medical teams of the Angiology Department. The clinical management of patients was instead in charge to the medical team of the ICU, including treatment and/or prophylaxis of venous thromboembolism (VTE), according to the SOC. The SOC consisted in VTE prophylaxis with low molecular weight heparin (LMWH) in every patient admitted to ICU, if not contraindicated. Anticoagulant dosage of LMWH (or other anticoagulant drugs) were only employed upon specific indications. A US examination could be required by the medical team of the ICU, usually for patients with high bleeding risk or for patients with clinical suspicion of VTE (Supplementary Fi 2). The SOC is in line with current international guidelines [9]. Medical teams in charge of US examinations were blinded to clinical data and pharmacological therapies. Data were then collected and analyzed by two authors (SM and VZ), not involved either in US examination or in the clinical management of patients.

### Collected data

US examination was performed by expert physicians in the field of vascular ultrasound, using a commercially available ultrasound system and 5.0–15.0 MHz linear probe (MyLab 50; Esaote, Genoa, Italy). The examinations consisted of a comprehensive B-mode ultrasound protocol, from thigh to ankle, employing compression and color-Doppler at selected sites, according to the Consensus Conference of the Society of Radiologists in Ultrasound [8]. DVTs were classified, according to the anatomical site, in proximal, distal or muscular. Proximal DVTs were located in the femoral–popliteal axis or in the great saphenous vein, within 5 cm from the saphenous–femoral crosse; distal DVTs were located in the main veins of the calf (tibial or peroneal) and, ultimately, muscular DVTs were located in the small muscular vessels of the calf [10, 11]. Illustrative pictures of US findings in DVT of lower limbs are reported in Supplementary Fig. 3.

Extension of a previously diagnosed DVT was defined as a longitudinal increase of a pre-existing thrombus toward a more proximal vessel (e.g., from distal calf to the popliteal vein), as well as the formation of a new thrombus in another vessel at the same level (e.g., on the contralateral side).

Clinical, hematological and biochemical data were collected from the medical records.

### Endpoints adjudication and sample size estimation

The primary endpoint of the trial was comparing the incidence of DVT in the two study groups. The secondary endpoint was the need for prophylaxis/treatment of the VTE.

We conducted then a post-hoc analysis, evaluating the occurrence of pulmonary embolism, major bleedings (as defined by the International Society of Thrombosis and Hemostasis [12, 13]), the occurrence of anemia the duration of ICU stay, and the risk of death in ICU.

Anemia was defined as a reduction of hemoglobin ≥ 2 g/dL during the ICU stay, without evidence of active bleeding, or necessity of concentrated red cells transfusion during the ICU hospitalization (intraoperative transfusions are excluded).

Based on existing literature [14, 15], we estimated an absolute difference in incidence of 0.25 in our primary endpoint. Assuming a type I error probability < 0.05, a sample size of 50 subjects per group allows a statistical potency of 90%.

### Statistical analysis

Continuous variables are expressed as mean (SD), for normally distributed variables, and as median [IQR] for non-normally distributed ones. Categorical variables are expressed as absolute and relative (%) frequencies. A significant difference was set for a probability of type I error < 0.05 under assumption of null hypothesis. Normality of distributions has been tested with the Kolmogorov–Smirnov test. Significance of differences has been tested with the Student’s *t* test and the Mann–Whitney *U* test for normally and non-normally distributed parameters, respectively. Differences in proportions were tested with the *χ*^2^ test. The Spearman rank test was employed to calculate non-parametric correlation coefficients. Statistical analysis was performed using SPSS package software v.27 (IBM, Armonk, NY). Graphs have been produced with Graph Pad Prism 9 (GraphPad Software, San Diego, CA).

## Results

Characteristics of the enrolled subjects, according to the inclusion in the screening and control group, are summarized in Table 1. Data were analyzed for all patients in both groups. Causes of admission to the ICU were comparable between the two groups. Similarly, median severity, expressed as SOFA and APACHE-II score, and risk of DVT, expressed as Padua score, were not significantly different between the two groups. Females were under-represented in the whole population, without differences between the two groups.

**Table 1:** Baseline characteristics of enrolled subjects at the admission to the intensive care unit (ICU)

Parameter	Screening (*n* = 50)	No screening (*n* = 50)	*p* value
Age (yrs), median [IQR]	56.8 (38.5–73.1)	58.9 (42.8–75.3)	0.519
Female sex, *n* (%)	14 (28.0)	17 (34.0)	0.666
Weight (kg), median [IQR]	70.0 (65.0–82.3)	77.0 (65.0–87.5)	0.474
Cause of admission to the ICU			
Cerebrovascular diseases, *n* (%)	21 (42.0)	14 (28.0)	0.208
Cardio-respiratory arrest, *n* (%)	2 (4.0)	3 (6.0)	0.999
Post-operatory, *n* (%)	8 (16.0)	15 (30.0)	0.153
Respiratory failure, *n* (%)	6 (12.0)	5 (10.0)	0.999
Trauma, *n* (%)	17 (34.0)	12 (24.0)	0.387
Other, *n* (%)	7 (14.0)	13 (26.0)	0.211
Glasgow coma scale, median [IQR]	7.0 (5.0–12.0)	7.0 (5.0–13.5)	0.256
Mean arterial pressure (mmHg)	80.0 (19.0)	78.4 (19.7)	0.661
Heart rate (bpm)	80.1 (23.7)	85.8 (20.8)	0.210
Respiratory rate (apm)	15.2 (3.4)	16.7 (7.9)	0.215
Body temperature (°C)	36.1 (1.1)	35.9 (0.9)	0.344
P/F ratio, median [IQR]	264 (182–363)	255 (173–346)	0.463
Hemoglobin (g/dL)	12.6 (2.0)	12.2 (1.9)	0.323
Platelets (× 10^6^/mL)	221.3 (73.4)	205.8 (100.0)	0.380
INR	1.1 (0.3)	1.2 (0.3)	0.176
White blood cells (× 10^3^/mL)	14.6 (6.1)	15.0 (6.6)	0.713
C-Reactive protein (mg/dL)	13.8 (11.8)	14.1 (12.3)	0.910
Creatinine (mg/dL)	1.1 (0.7)	1.1 (0.6)	0.651
eGFR (mL/min)	84.9 (31.0)	78.3 (31.7)	0.298
APACHE-II score, median [IQR]	15.5 (11.0–22.0)	13 (10.0–19.3)	0.263
APACHE-II estimated risk (%), median [IQR]	15.0 (12.0–36.3)	13.5 (7.0–26.3)	0.093
SOFA score, median [IQR]	5.5 (4.0–.0)	6.0 (3.0–9.0)	0.413
Padua score, median [IQR]	5.0 (5.0–6.0)	6.0 (5.0–7.0)	0.083

*INR* international normalized ratio, *eGRF* estimated glomerular filtration rate, *APACHE-II* acute physiology and chronic health disease classification system II, *SOFA* sequential organ failure assessment

### Ultrasound (US) examination findings

**Fig. 1:**
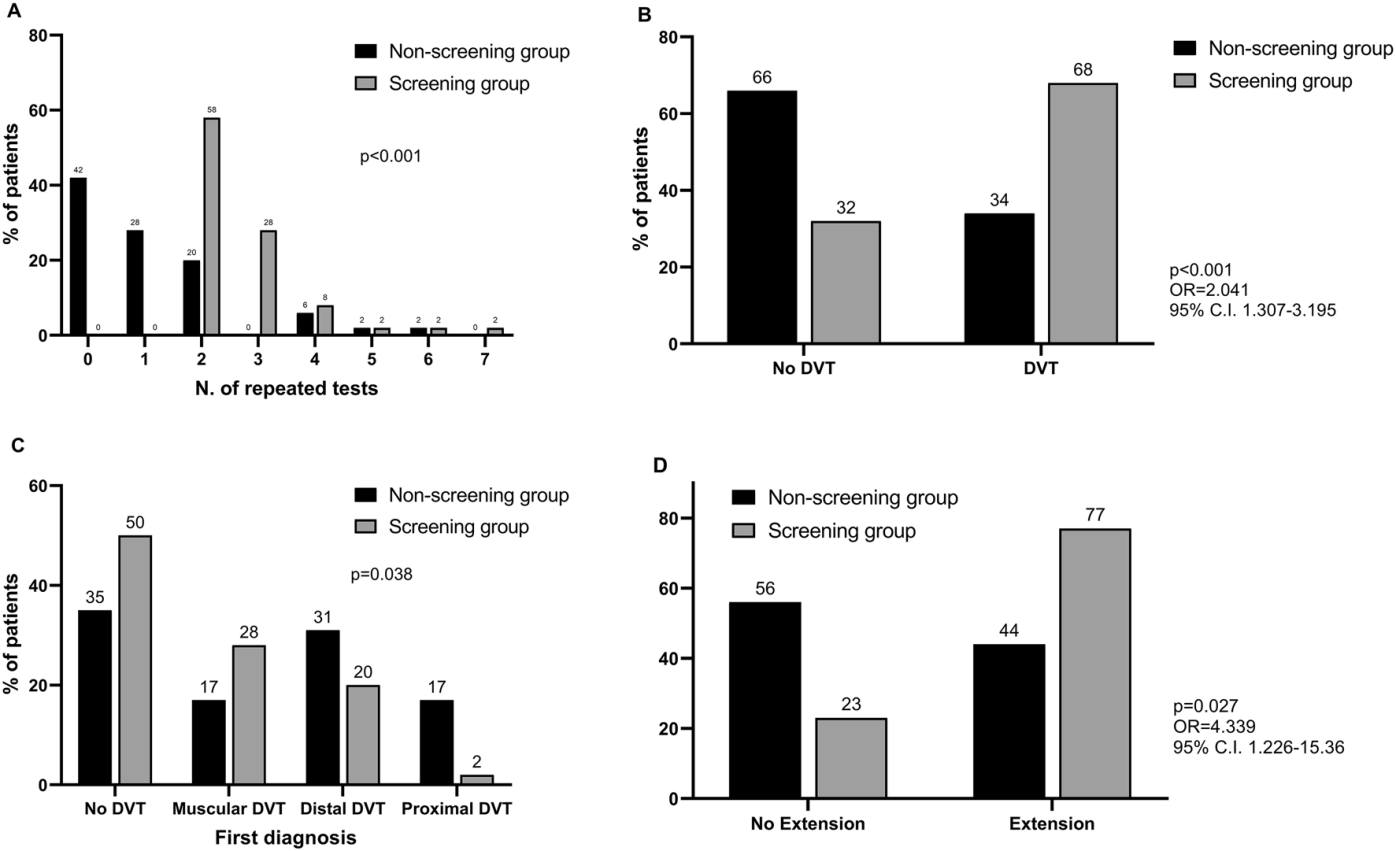
**A** Number of color-Doppler ultrasound (CDU) examinations of lower limbs performed in the two groups of subjects. **B** Prevalence of deep vein thrombosis (DVT) in the two group of subjects. **C** Results of the first color-Doppler ultrasound examinations in screening and non-screening group. **D** Prevalence of extending thrombosis in the two groups

All patients in the screening group underwent at least 2 US examinations of lower limbs veins up to a maximum of 7 exams (median 2, IQR 2–3). Most of patients in the non-screening group did not undergo any US examination (median 1, IQR 0–2) (Fig. 1A). Overall, lower limbs DVT was significantly more frequent in the screening compared to the non-screening group with an OR of 2.041 (95% C.I. 1.307–3.195; Fig. 1B). However, the non-screening group was characterized by increased incidence of both distal and proximal DVT compared to the screening group (Fig. 1C). When overtime extension of pre-existing DVT in subsequent examinations was considered, this event was significantly more frequent in the screening group (OR 4.339, 95% C.I. 1.226–15.36; Fig. 1D). More specifically, extension to the proximal vein tract was observed in 3 patients, one in the screening and two in the non-screening group, respectively. In the screening group, the first US examination was negative in 25 patients (50.0%), whereas 9 of them (18.0%, 95% C.I. 7.1–28.9%) had a diagnosis of distal DVT in subsequent examinations. No proximal DVT was observed in patients with a first negative examination.

### Secondary outcome

**Fig. 2:**
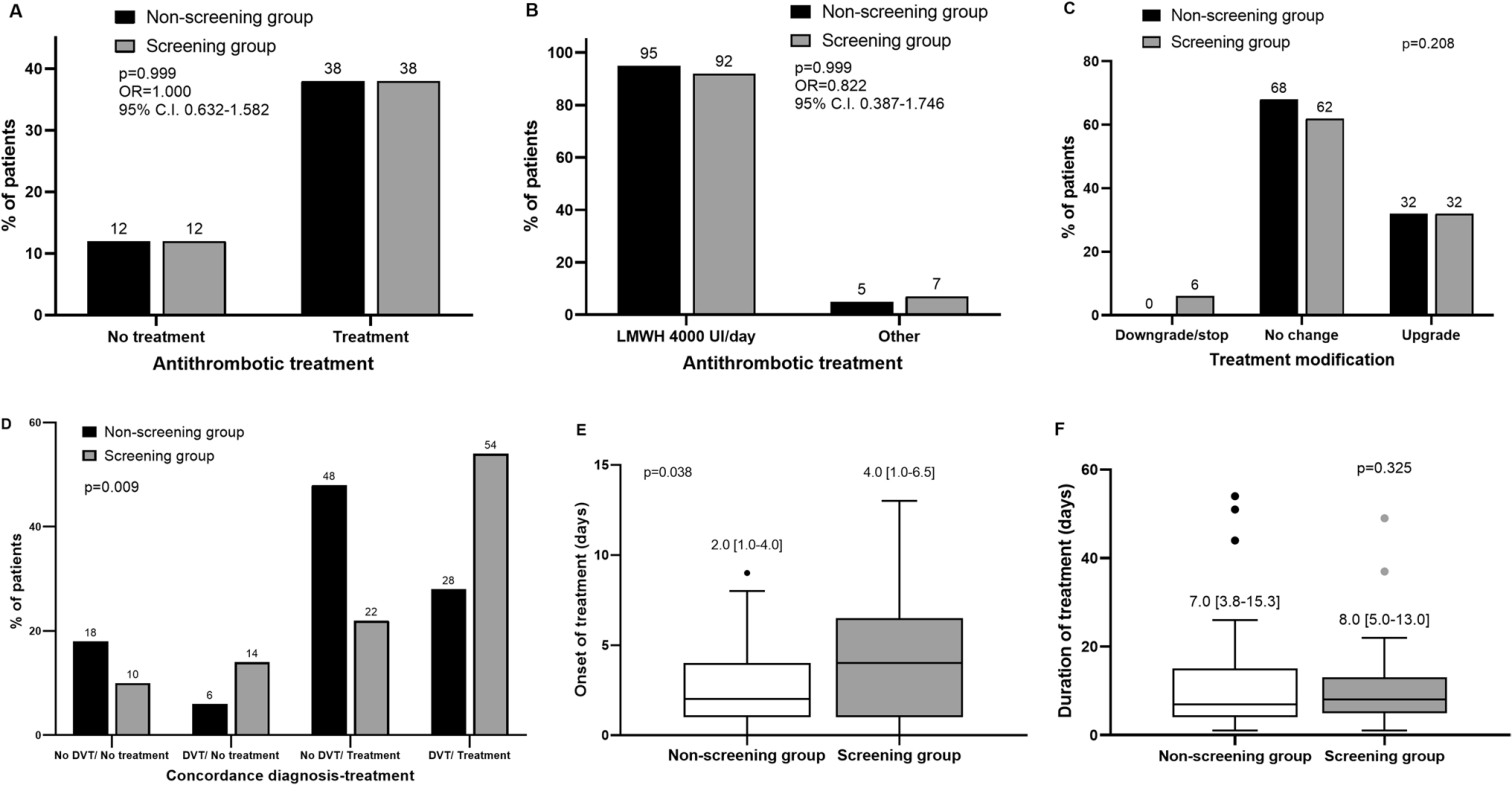
**A** Prevalence of pharmacological antithrombotic treatment in the two groups.﻿ **B** Type of pharmacological treatment and relative dosage in the two groups.P **C** Therapeutic modifications during the stay in intensive care unit (ICU) in the two groups. **D** Concordance of the ultrasound diagnosis of deep vein thrombosis (DVT) and the associated pharmacological treatment in the two groups.  **E** Overall duration of the pharmacological antithrombotic treatment during the stay in ICU, in the two groups. **F** Time between the admission and the initiation of antithrombotic pharmacological treatment in the two groups. LMWH: low-molecular weight heparin

Seventy-six patients (76%) received pharmacological antithrombotic therapy. The use of antithrombotic therapy was not significantly different in the two groups (Fig. 2A). All treated patients received low-molecular weight heparin (LMWH, enoxaparin). The most common initial treatment was LMWH with prophylactic dosage (Fig. 2B). Thirty-five patients (35%) underwent a modification of therapy during the hospital stay, with no significant difference between screening and non-screening group (Fig. 2C); of them, only three patients experienced a downgrading of the treatment and they were all in the screening group. Figure 2D displays the concordance between the diagnosis of DVT and the use of antithrombotic drugs: we observed a significant increase of positive concordance (presence of DVT, treated), with a specular reduction of negative concordance (absence of DVT, untreated).

Patients in the screening group started the antithrombotic treatment significantly later (Fig. 2E), although the median duration of the treatment showed no significant difference between the two groups (Fig. 2F). We analyzed the potential determinants of this delay in the screening group (Table 2) and, although the majority of correlations were shared the two groups (e.g., respiratory failure reduces the time to onset of the antithrombotic treatment in both groups), some differences can be observed: factors that reduce the time to onset in the non-screening group (e.g., age, Glasgow Coma Scale and *P*/*F* ratio) are not significant in the screening group, whereas other factors delaying the onset of the antithrombotic treatment in the screening group (e.g., cerebrovascular diseases) are not significant in the non-screening group.

**Table 2:** Potential determinants of delayed antithrombotic treatment onset in the overall cohort and in the two groups. Significant correlations are marked with *.

Parameters	Overall cohort (*n* = 80)		Non-screening group (*n* = 39)		Screening group (*n* = 41)	
	ρ	*p*	ρ	*p*	ρ	*p*
Age	− 0.277	0.013*	− 0.427	0.007*	− 0.171	0.285
Cause of admission to the ICU						
Cerebrovascular diseases	0.455	< 0.001*	0.272	0.094	0.541	< 0.001*
cardio-respiratory arrest	− 0.114	0.313	0.047	0.775	− 0.222	0.162
Post-operatory	− 0.116	0.307	− 0.022	0.894	− 0.168	0.294
Respiratory failure	− 0.420	< 0.001*	− 0.430	0.006*	− 0.457	0.003*
Trauma	0.273	0.014*	0.216	0.186	0.271	0.087
Other	− 0.299	0.007*	− 0.299	0.065	− 0.230	0.148
Glasgow coma scale	− 0.117	0.301	− 0.317	0.050*	0.130	0.419
Mean arterial pressure	0.260	0.020*	0.240	0.140	0.285	0.071
Heart rate	− 0.264	0.018*	0.334	0.159	− 0.282	0.074
Respiratory rate	− 0.237	0.034*	− 0.270	0.096	− 0.278	0.079
Body temperature	− 0.057	0.615	− 0.069	0.676	− 0.138	0.395
P/F Ratio	0.277	0.013*	0.394	0.013*	− 0.151	0.346
Hemoglobin	0.176	0.119	0.045	0.786	0.186	0.245
Platelets	− 0.066	0.562	− 0.273	0.092	− 0.006	0.971
INR	− 0.241	0.032*	0.012	0.944	− 0.288	0.068
White blood cells	− 0.025	0.826	− 0.163	0.321	0.057	0.724
C-Reactive protein	− 0.125	0.285	− 0.017	0.925	− 0.176	0.370
Creatinine	− 0.292	0.009*	− 0.206	0.209	− 0.303	0.054
eGFR	0.349	0.002*	0.271	0.095	0.362	0.020*
APACHE-II score	− 0.139	0.220	− 0.018	0.915	− 0.305	0.053
SOFA score	− 0.184	0.102	0.124	0.453	− 0.423	0.006*
Padua score	− 0.103	0.366	0.169	0.303	− 0.185	0.248

Six patients (6%) were positioned an inferior vena cava filter, 3 in the screening group and 3 in the non-screening group.

Four out of 6 patients with a diagnosis of proximal DVT were treated with full dose of LMWH (100 IU/kg/day), whereas 2 patients were treated with intermediate dose, due to relative contraindications.

Patients with a diagnosis of muscular or distal DVT were mainly treated with LMWH with prophylactic dosage (53%), and only 23% of them was treated with intermediate or full anticoagulant dosage. As shown in Supplementary Fig. 4, patients in the screening group receiving a diagnosis of distal/muscular DVT were more likely to receive a full anticoagulant treatment. One patient in the screening group was positioned an inferior vena cava filter.

### Post-hoc analysis

**Fig. 3:**
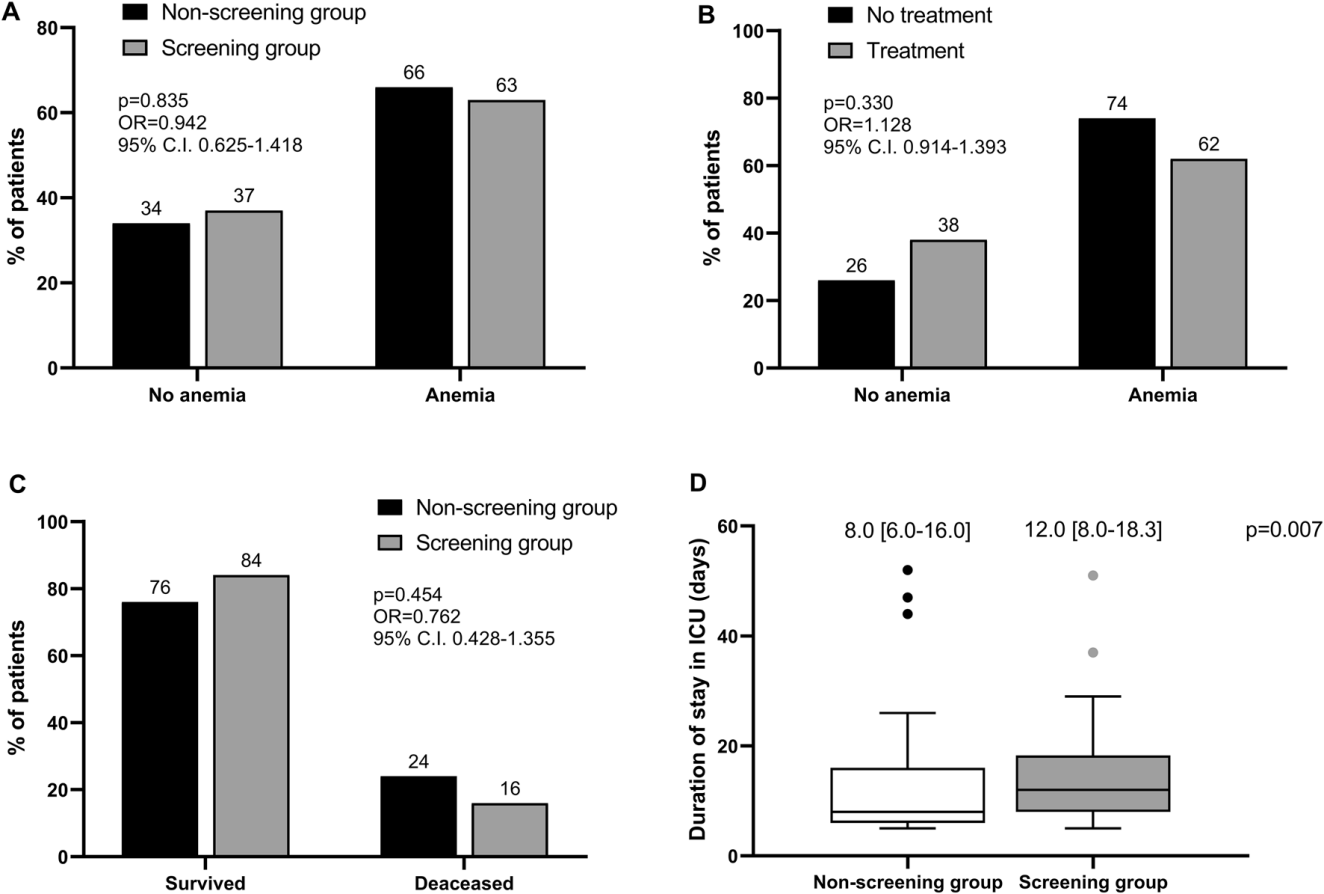
**A** Incidence of clinically relevant anemia in the two groups. **B** Incidence of clinically relevant anemia in patients treated and not treated with antithrombotic drugs. **C** Risk of death during the stay in Intensive Care Unit (ICU) in the two groups. **D** Duration of stay in the ICU in the two groups

Pulmonary embolism was diagnosed through contrast enhanced chest CT scan in 7 patients (7%), 3 (6%) in the screening group and 4 (8%) of the non-screening group (*p* > 0.05). Major bleedings were reported in 5 patients (5%), 4 (8%) in the non-screening group and in 1 (2%) in the screening group (*p* > 0.05). Anemia was reported in 64 patients (64.0%), without significant differences between the two groups (Fig. 3A). The incidence of anemia was higher in patients treated with antithrombotic drugs, although in a non-significant manner (Fig. 3B). No significant difference was observed in the risk of death in ICU (Fig. 3C), whereas the duration of stay in ICU was significantly longer in the screening group (Fig. 3D).

## Discussion

The results of this pilot trial show that the screening program for DVT is associated with an increased number of DVT diagnoses. Existing data report an incidence of DVT among critically ill patients ranging between 5 and 30%, depending on the reporting methods [3, 14–16]. We observed a 68% incidence of DVT in the screening group, suggesting that the incidence of DVT in critically ill patients is currently underestimated. The majority of DVTs in the screening group were localized at muscular or distal level. Clinical relevance of these DVTs is still matter of debate [17, 18]. On the other hand, proximal DVTs were mostly diagnosed in the non-screening group, and this could be due to an early diagnosis of distal DVTs in the screening group. Indeed, patients in the screening group receiving a diagnosis of distal/muscular DVT were more likely to receive a full anticoagulant treatment. Therefore, we hypothesize that early diagnosis of distal DVT may lead to therapeutic adjustments, which eventually help preventing the extension of distal DVTs to proximal veins.

Although extension of a pre-existing DVT was more frequent in the screening group, this can be due to the general increase of diagnosis rate. Overall, the extension to the proximal veins was uncommon (3% in the whole cohort). These findings are in line with previous studies, estimating an incidence of progression for a distal DVT toward the proximal district of 1.7% after 6 weeks in patients treated with LMWH [18], and 6.3% after 6 weeks in untreated patients [19]. Compared to previous studies, we employed a stricter definition of DVT extension, including the extension from small muscular veins to large distal veins, and the formation of a new thrombus in another vessel at the same level. To our knowledge, no previous study reported the risk of extension of muscular or distal DVTs in the same level.

The risk of finding an incident new DVT after a first negative US examination was also quite low (18%), and none of them was a proximal DVT. This result is in line with previous findings, especially with the large observational study of Loffredo et al. [20], showing that 90% of asymptomatic DVTs in acutely ill patients are found within the first 48 h from admission. Although the two studies have significant differences in setting (internal medicine ward *vs* ICU) and methods (compression ultrasound *vs* complete US), our study supports the evidence that asymptomatic DVTs in acutely ill patients occur in the early hours after admission.

Considering the low risk of proximal extension distal DVTs and the low risk of finding an incident DVT after a first negative examination, our results are insufficient to support the routine repetition of the US examination.

The increased number of DVT diagnoses in the screening group was not associated with an increased treatment, in terms of number of treated patients, dosage and duration of the treatment. Moreover, 3 patients in the screening group experienced a reduction of the treatment. The screening was not associated with an increased incidence of anemia or major bleedings, in our observation time.

The screening was associated with an increased concordance between diagnosis and treatment and a delayed initiation of the antithrombotic treatment. As highlighted by results in Table 2, the latter aspect could be particularly relevant in patients with increased bleeding risk of potentially treatable or reversible cause (e.g., polytrauma, intracranial bleedings, etc.) From a general point of view, our results suggest that the timing of starting the anti-thrombotic therapy is mainly influenced by risk factors for VTE in the non-screening group, whereas the risk factors for bleeding have a larger weight in the screening group.

On the other hand, the screening is associated to an increased number of US examinations and a prolonged duration of stay in ICU, contributing to an increased cost for the hospitalization. A longer stay in ICU could be also due to a higher survival rate, although this result was not statistically significant in our study.

In summary, the US screening for DVTs is associated to an increased diagnosis of distal and muscular DVTs, mainly within 48 h from the admission. It also associated with a reduced incidence of proximal DVTs. The screening did not have a net effect on dosage and duration of the anti-thrombotic therapy and it may lead to increased healthcare costs. However, it could have a profitable cost/benefit profile in specific subsets of patients, like those with an increased bleeding risk. Future larger studies are needed to investigate the effects of the screening on survival rate and to define the cost/benefit profile of the screening.

The current study is the first head-to-head trial testing systematic DVT screening; furthermore, it is the first trial employing a comprehensive US examination, including Doppler, instead of the simple CUS. However, we must acknowledge the limitations of the present study: first of all, as a monocentric study, the trial could not be completely randomized or blinded. Furthermore, the patients were not randomly allocated in the study groups, but consecutively. Being the patients enrolled in one single ICU, this approach was chosen to minimize the possible reciprocal interference between the two arms. To achieve a completely randomized and blinded study, a larger multi-centric trial is needed. Second, the trial was retrospectively registered after completion of the enrollment. Ultimately, the small sample size prevented us from achieving statistical significance for hard endpoints, such as the risk of pulmonary embolism, major bleeding and death. As a consequence, this trial should be considered as a pilot study and the results should be considered as preliminary.

## Conclusions

The results of the present trial suggest that active screening for DVT with complete US examination of the lower limbs is associated with an increased diagnosis of DVT, without a corresponding overtreatment. The screening could be associated with a reduced incidence of proximal DVT and could improve the management of the patients with increased bleeding risk.

Larger studies are needed to confirm our results, to optimize the early diagnosis and the overall management of the venous thromboembolism in critically ill patients.

## Supplementary Information

Below is the link to the electronic supplementary material.

Supplementary file1 (DOCX 1180 KB)

Supplementary file2 (DOC 221 KB)

## Data Availability

The data sets used during the current study are available from the corresponding author on reasonable request.

## Abbreviations

CT: Computed tomography
CUS: Compression ultrasound
DVT: Deep vein thrombosis
ICU: Intensive care unit
SOC: Standard of care
VTE: Venous thromboembolism
