# Supplementary material for: Ultrasound screening for asymptomatic deep vein thrombosis in critically ill patients: a pilot trial
Source: Intern Emerg Med. 2022 Aug 31;17(8):2269–77. doi: 10.1007/s11739-022-03085-8 (PMC9428380; doi:10.1007/s11739-022-03085-8)

**Supplementary Figures**

**Supplementary Figure 1.** Flow-chart of subjects’ enrollment in the trial.

**
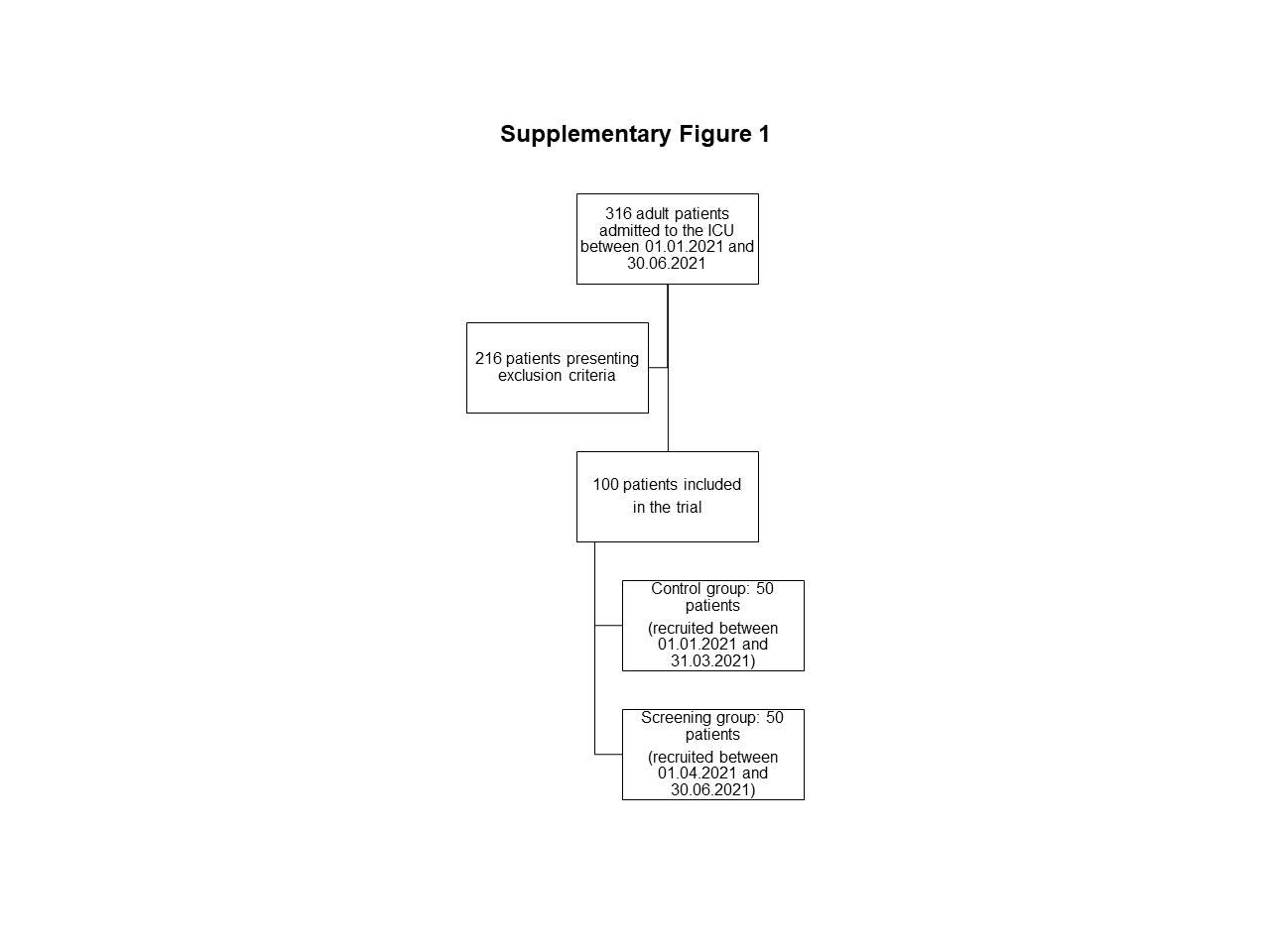
**

**Supplementary Figure 2.** Visual summary of the study design.


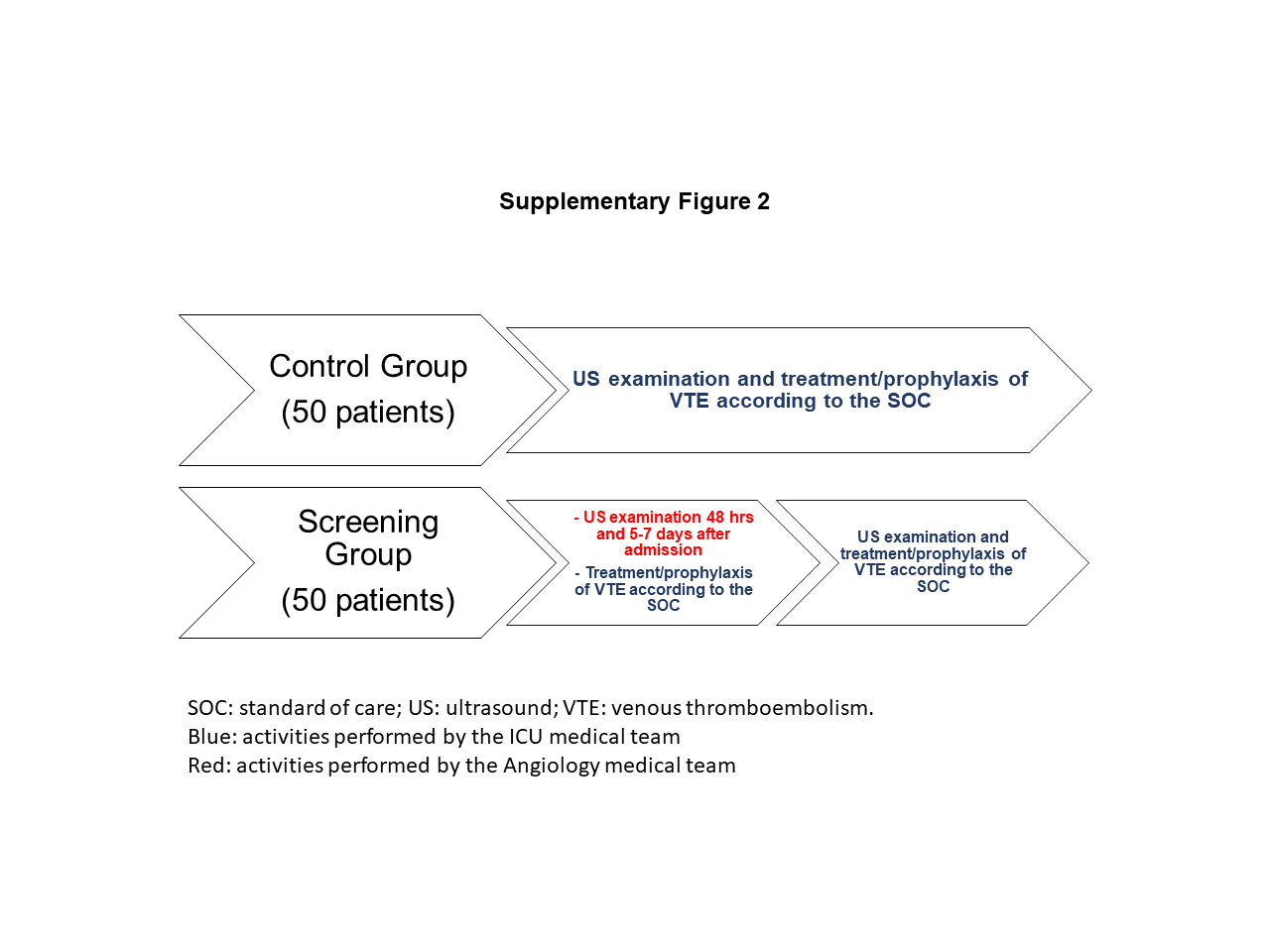


**Supplementary Figure 3.** Illustrative pictures of DVT of the lower limb US. **A.** Transversal scan at the proximal hindlimb. **B.** Longitudinal scan at the proximal hindlimb.


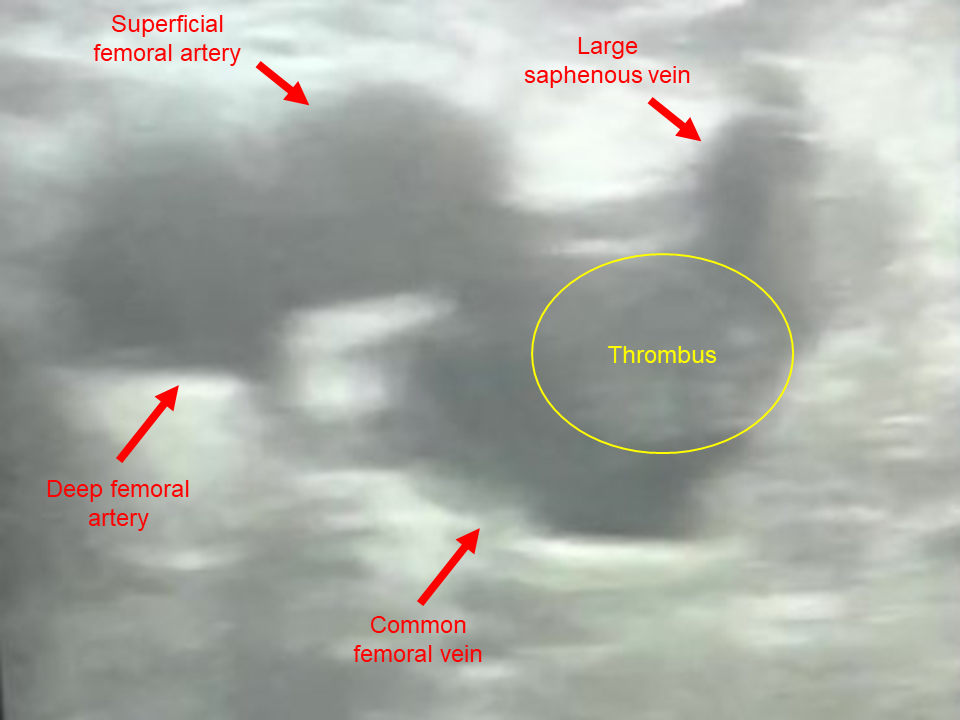

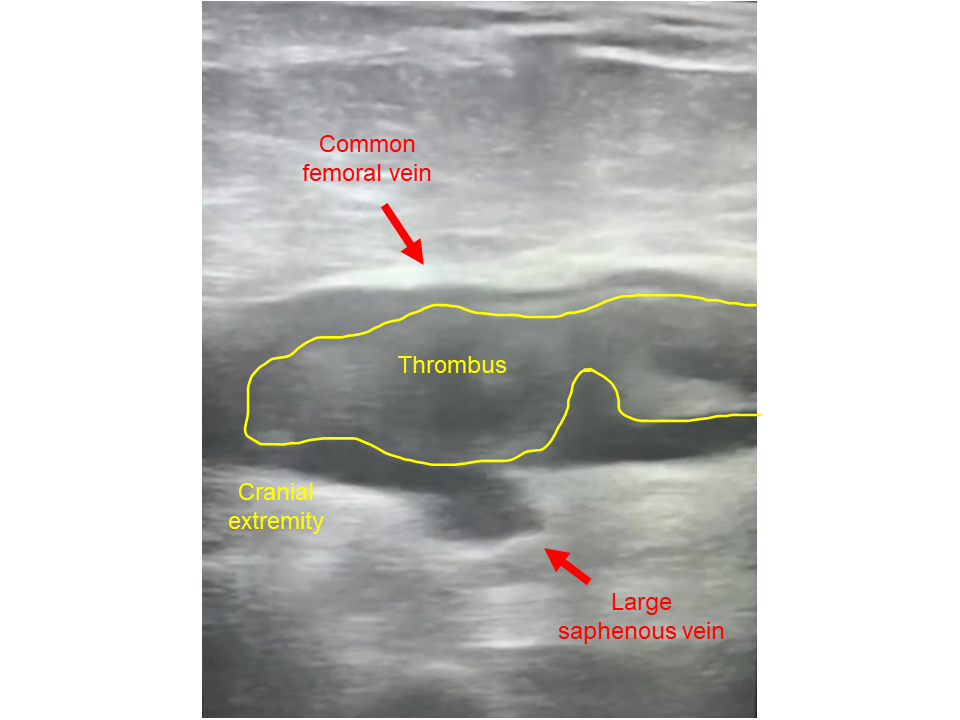


B

A

**Supplementary Figure 4.** Pharmacological antithrombotic treatment of patients receiving a diagnosis of distal or muscular deep vein thrombosis (DVT). **A.** In the non-screening group. **B.** In the screening group. **C.** Comparison of proportions of patients receiving full anticoagulant treatment after a diagnosis of distal or muscular DVT. LMWH: low-molecular weight heparin. Low: 4000 IU/day. Full: 100 IU/kg/day. Intermediate: any dosage between low and full.


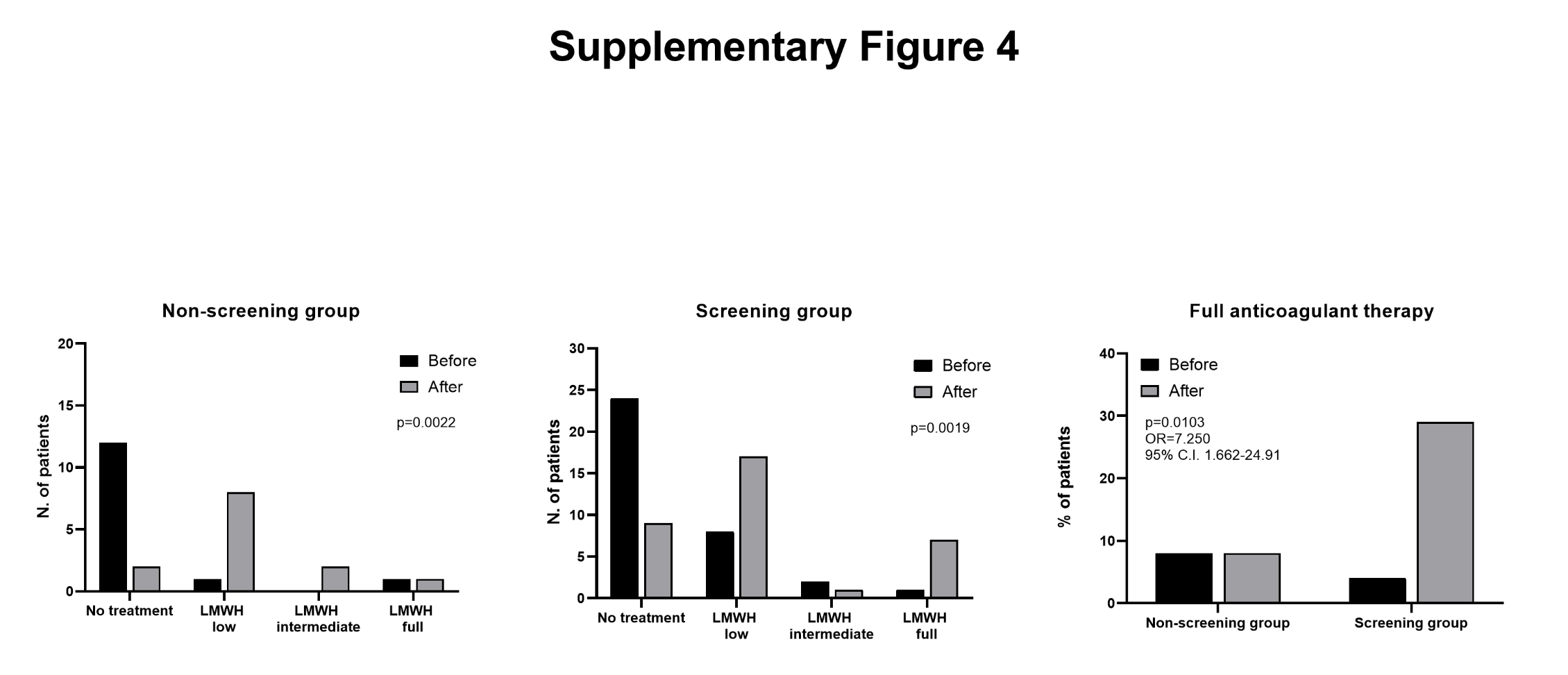

Supplement: Supplementary file 1 — Supplementary file1 (DOCX 1180 KB) [file 11739_2022_3085_MOESM1_ESM.docx]
